# Different particle determinants induce apoptosis and cytokine release in primary alveolar macrophage cultures

**DOI:** 10.1186/1743-8977-3-10

**Published:** 2006-06-14

**Authors:** Magne Refsnes, Ragna B Hetland, Johan Øvrevik, Idunn Sundfør, Per E Schwarze, Marit Låg

**Affiliations:** 1 Department of Air pollution and Noise, Division of Environmental Medicine, Norwegian Institute of Public Health, P.O. Box 4404 Nydalen, NO-0403 Oslo, Norway

## Abstract

**Background:**

Particles are known to induce both cytokine release (MIP-2, TNF-α), a reduction in cell viability and an increased apoptosis in alveolar macrophages. To examine whether these responses are triggered by the same particle determinants, alveolar macrophages were exposed *in vitro *to mineral particles of different physical-chemical properties.

**Results:**

The crystalline particles of the different stone types mylonite, gabbro, basalt, feldspar, quartz, hornfels and fine grain syenite porphyr (porphyr), with a relatively equal size distribution (≤ 10 μm), but different chemical/mineral composition, all induced low and relatively similar levels of apoptosis. In contrast, mylonite and gabbro induced a marked MIP-2 response compared to the other particles. For particles of smaller size, quartz (≤ 2 μm) seemed to induce a somewhat stronger apoptotic response than even smaller quartz (≤ 0.5 μm) and larger quartz (≤ 10 μm) in relation to surface area, and was more potent than hornfels and porphyr (≤ 2 μm). The reduction in cell viability induced by quartz of the different sizes was roughly similar when adjusted to surface area. With respect to cytokines, the release was more marked after exposure to quartz ≤ 0.5 μm than to quartz ≤ 2 μm and ≤ 10 μm. Furthermore, hornfels (≤ 2 μm) was more potent than the corresponding hornfels (≤ 10 μm) and quartz (≤ 2 μm) to induce cytokine responses. Pre-treatment of hornfels and quartz particles ≤ 2 μm with aluminium lactate, to diminish the surface reactivity, did significantly reduce the MIP-2 response to hornfels. In contrast, the apoptotic responses to the particles were not affected.

**Conclusion:**

These results indicate that different determinants of mineral/stone particles are critical for inducing cytokine responses, reduction in cell viability and apoptosis in alveolar macrophages. The data suggest that the particle surface reactivity was critical for cytokine responses, but contributed less to cell death for the types of particles tested. The size-dependent variations, specially in cytokine release, seem not to be explained only by particle surface area.

## Background

A wide range of different particles from ambient air and occupational settings is known to elicit inflammatory processes and to induce cell death in the lung [1-3]. Inflammation is an important protective response, but may also be involved in triggering acute adverse health effects and development of chronic lung disease [1,4]. Furthermore, apoptotic and necrotic cell death seem to be important denominators in respiratory disease, and may contribute both in acute lung injury and in development of chronic lung disease [5,6].

Alveolar macrophages are major effector cells of the non-specific host defence in the lung. The early responses by the macrophages to inhaled foreign material, including airborne particles and pathogens, are essential for a proper activation of the pulmonary immune system, and seem to play a key role in the response to toxicants [1,7]. These responses involve the production/release of cytokines that are crucial for inflammatory processes [8]. Proinflammatory cytokines from alveolar macrophages, such as tumour necrosis factor (TNF)-α and interleukin (IL)-1β, enhance the release of chemokines from lung epithelial cells, leading to recruitment of neutrophilic cells to the sites of cell injury [1,9,10]. Among the rat chemokines, macrophage inflammatory protein (MIP)-2 appears to be critical in lung inflammatory responses [8,11].

Macrophages undergo both apoptotic and necrotic cell death in response to noxious stimuli. Since viable macrophages play a crucial role in the clearance of inhaled particles and cellular debris from the alveolar region, the cytotoxic response of macrophages may contribute to the final outcome of the toxicant exposure. It has been demonstrated that several types of particles, including quartz [12-14], urban air particles and diesel exhaust particles [15,16] induce apoptosis in macrophages. The balance between cytokine induction and different types of cell death (apoptosis, necrosis) may be important for the outcome of particle exposure, and the development of both acute and chronic disease, but these processes are far from clarified.

Accumulating evidence suggests that small particles are more potent than larger particles, due to both their deposition pattern and large surface area to mass ratio [17]. Several studies show that correlating for differences in total surface area may adjust for differences in biological reactivity among particles of similar composition [18-22]. However, differences in surface area appear to be insufficient to account for potency variations between different mineral particles [22]. For mineral particles the crystalline structure and surface reactivity, and not the leachable components, seem to be the crucial determinants for cellular responses [22-26], whereas for combustion particles both particle core and leachable constituents (metals, organic compounds) seem to be involved [15,16].

An important question is whether the induction of different end-points is mediated by similar particle characteristics. Although the ability of many particles to induce the release of different cytokines and cell death (apoptosis/necrosis) has been described, the studies are difficult to compare because of variations in the test systems. Most importantly, few studies have focused on the effects on both cytokine release and cell death, and there are few comparative studies on particles of varying characteristics. In the present study the ability of mineral particles of various sizes and surface areas, structures (crystalline and amorphous) and chemical composition to induce changes in apoptosis/cell viability versus cytokine responses in primary rat lung macrophages is examined, to delineate the importance of particle characteristics for different biological end-points.

## Results

**Figure 1:**
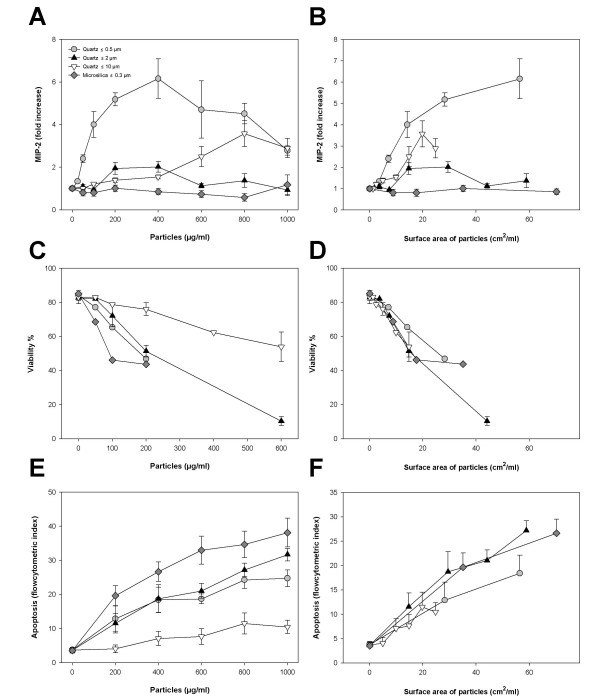
**MIP-2 response and cell death in rat alveolar macrophages after exposure to quartz particles of different sizes and to amorphous microsilica**. The macrophages were exposed to quartz (≤ 0.5 μm, ≤ 2.0 μm, ≤ 10 μm) and microsilica (≤ 0.3 μm) for 20 h as described in Materials and Methods. **A, B: **MIP-2, concentration-effect curve. **C, D: **Cell viability, concentration-effect curve. **E, F: **Apoptosis, concentration-effect curve. In **A, C, E **the data are related to the mass of the particles, and in **B, D, F **to the surface area of the particles. The MIP-2 responses were analysed by ELISA. The cell viability was measured by exclusion of PI in a fluorescence microscope. The apoptosis was analysed by flow cytometry after staining by Hoechst 33258. The results represent the mean +/- SEM of three independent experiments.

The ability of different particles to induce cytokine release and apoptosis in rat primary alveolar macrophages was examined. Table 1 shows the particle sizes and surface areas of the mineral particles used in this study. Alveolar macrophages were exposed to quartz of different sizes, quartz-0.5 (≤ 0.5 μm), quartz-2 (≤ 2 μm), quartz-10 (≤ 10 μm), and to amorphous microsilica (≤ 0.3 μm) for 20 h and analysed for release of the chemokine MIP-2, cell viability and apoptosis (Fig. 1). Fig. 1A shows the MIP-2 release, as a function of particle mass, in a concentration range from 25–800 μg/ml. Quartz-0.5 induced a marked MIP-2 response, with a six-fold increase at 400 μg/ml and a reduction at higher concentrations. The response to quartz-2 was less, with only about a two-fold increase at 200–400 μg/ml and lesser responses at higher concentrations. For quartz-10 the MIP-2 responses were increased up to 800 μg/ml, whereas for microsilica no significant changes in MIP-2 release were observed (Fig. 1A). Adjusting for differences in particle surface area could not account for differences in the MIP-2 release for all the particles (Fig. 1B). Most clearly, quartz-0.5 was markedly more potent than quartz-2.

**Table 1:** Sizes and surface areas of different stone particles

**Stone particles**	**Size fraction (μm)**	**Median diameter (μm)**	**Particle surface (m**^2^**/g)**
Quartz	≤ 0.5	0.4	14.1
	≤ 2	1.2	7.4
	≤ 10	8.0	2.5
Hornfels	≤ 2	1.8	15.0
	≤ 10	6.6	7.0
Fine grain syenite porphyr	≤ 2	1.3	12.2
	≤ 10	5.7	5.3
Microsilica	≤ 0.3	0.3	17.6
Gabbro	≤ 10	6.9	3.4
Mylonite	≤ 10	5.5	5.0
Basalt	≤ 10	5.0	7.0
Feldspar	≤ 10	5.6	3.6

**Figure 2:**
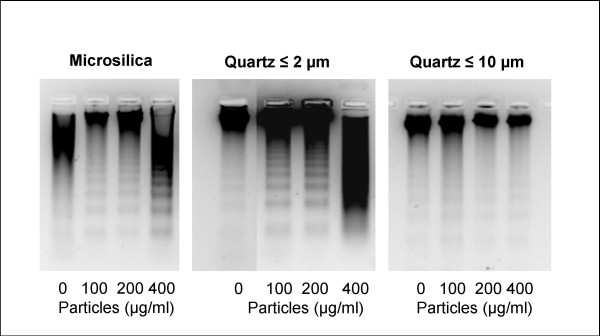
**DNA-fragmentation induced by quartz of different sizes and concentrations**. The macrophages were exposed to various quartz concentrations for 20 h. The DNA-fragmentation was measured as described in the Materials and Methods. The results represent a typical of three experiments.

With respect to the potential to reduce cell viability, the order was: microsilica > quartz-0.5 ~ quartz-2 > quartz-10, when related to particle mass (Fig. 1C). However, relative to particle surface areas the differences in reduction of cell viability were less pronounced (Fig. 1D). Thus, the particle surface area seemed to account for most of the observed differences in effects on cell viability. Fig. 1E shows the apoptotic response relative to mass concentration, as measured by flow cytometry. The exposure to quartz-2 and quartz-0.5 induced strong and roughly similar apoptotic responses, whereas quartz-10 showed lower responses. Microsilica elicited an even greater response than the quartz particles. As with the effects on cell viability, adjustments for differences in particle surface area seemed to account for most of the observed differences in particle-induced apoptosis (Fig. 1E, 1F), although quartz-2 might seem somewhat more potent than quartz-0.5 and quartz-10. The ability to induce apoptosis was confirmed by DNA fragmentation after gel electrophoresis, as illustrated by the effects of increasing mass concentrations of quartz-2 and microsilica. The quartz-10 was less potent (Fig. 2).

**Figure 3:**
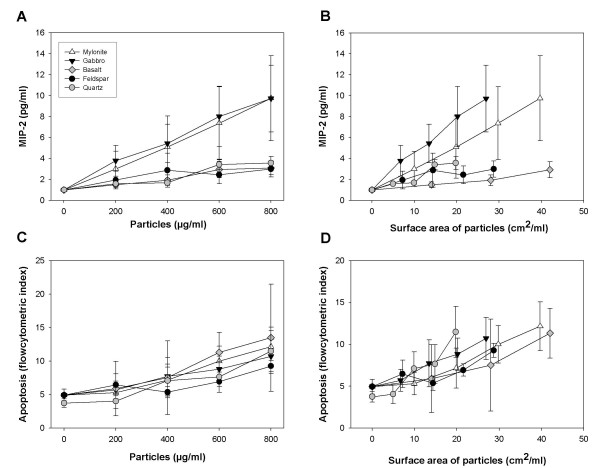
**MIP-2 response and apoptosis induced by various concentrations of stone particles**. The macrophages were exposed to mylonite, gabbro, feldspar, basalt and quartz (≤ 10 μm) for 20 h. **A, B) **show MIP-2 responses and **C, D) **Apoptosis. In **A **and **C **the MIP-2 responses and apoptosis, respectively, were related to the mass of the particles, and in **B **and **D **to the surface areas of the particles. The MIP-2 release was analysed by ELISA, and apoptosis by flow cytometry after Hoechst 33258 staining. The results represent the mean +/- SEM of three independent experiments.

The studies with pure quartz of various sizes and microsilica particles show a different pattern for induction of MIP-2 and cell death, suggesting that different particle determinants are involved in these processes. To corroborate this, stone particles (mylonite, gabbro, feldspar, basalt and quartz) of varying composition, but with similar size distribution (≤ 10 μm), were examined (Fig. 3). Very different MIP-2 responses were induced by these particles after 20 h of exposure, with mylonite and gabbro as the most marked stimulators on a mass basis (Fig. 3A). Basalt and feldspar exerted responses in the same range as quartz (Fig. 3A). As with the quartz and microsilica particles, differences in ability to induce MIP-2 release could not be attributed to differences in particle surface area. When related to particle surface areas, gabbro and mylonite were still most potent, and basalt least potent (Fig. 3B). The ability of these particles to induce apoptosis was relatively low, with apoptotic indexes of 8–13% at 800 μg/ml for all the mineral particles as measured by flow cytometry after 20 h of exposure, compared to ~ 5% in the controls. In contrast to the cytokine responses, the differences in apoptotic potentials were rather small and insignificant both when related to mass and particle surface area (Fig. 3C, 3D).

**Figure 4:**
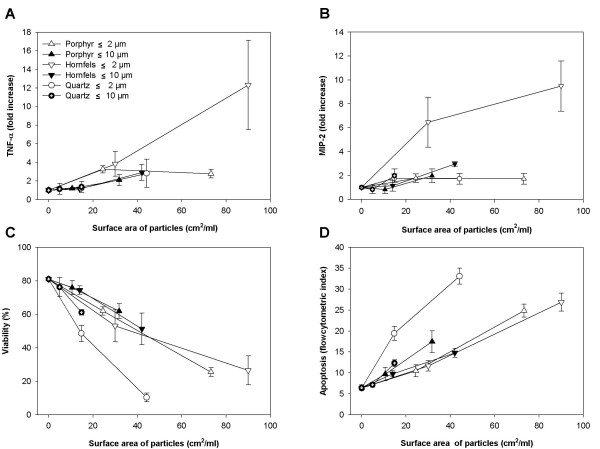
**Cytokine responses and cell death induced by hornfels and porphyr particles of different sizes: Comparison to quartz**. **A) **TNF-α responses, **B) **MIP-2 responses, **C) **Cell viability and **D) **Apoptosis. The macrophages were exposed to ≤ 2.0 μm and ≤ 10 μm particles of porphyr, hornfels and quartz for 8 h (TNF-α) or 20 h (MIP-2, viability and apoptosis). The cytokine release was analysed by ELISA, cell viability by the ability to exclude PI as assessed by fluoroscense microscopy and apoptosis by flow cytometry after Hoechst 33258 staining. The data are related to the surface area of the particles. The results represent the mean +/- SEM of three independent experiments.

To assess whether the apparently somewhat stronger ability of the quartz-2 fraction to induce apoptosis, and the low ability to trigger cytokine release, was peculiar to just this mineral, we examined the apoptotic and cytokine-releasing potential of the ≤ 2 μm fractions of two other mineral particles, porphyr and hornfels. Previous studies from our group have shown that porphyr induced very little MIP-2 release from primary rat type 2 alveolar epithelial cells, whereas hornfels elicited high levels of MIP-2 release from these cells [22]. The responses to different sizes of these particles and to quartz with similar size distributions were compared. TNF-α was measured in addition to MIP-2. The effects on cell viability are also presented, and all data are given in relation to particle surface area (Fig. 4). The results showed that porphyr-2, porphyr-10 and hornfels-10 induced only low levels of MIP-2 and TNF-α, whereas hornfels-2 elicited a strong increase in release of both cytokines from the macrophages (Fig. 4A, 4B). The effects of hornfels and porphyr on cell viability and apoptosis differed marginally, if at all (Fig. 4C, 4D). Thus, the data for hornfels and porphyr did not confirm the difference in apoptosis observed for quartz-2 and quartz-10 in Fig. 1 and also in Fig. 4. Notably, quartz-2 was more potent than the respective hornfels-2 and porphyr-2 fractions in inducing apoptosis and change in cell viability. Importantly, the pattern of induction of cytokines differed from the effects on apoptosis and cell viability.

**Figure 5:**
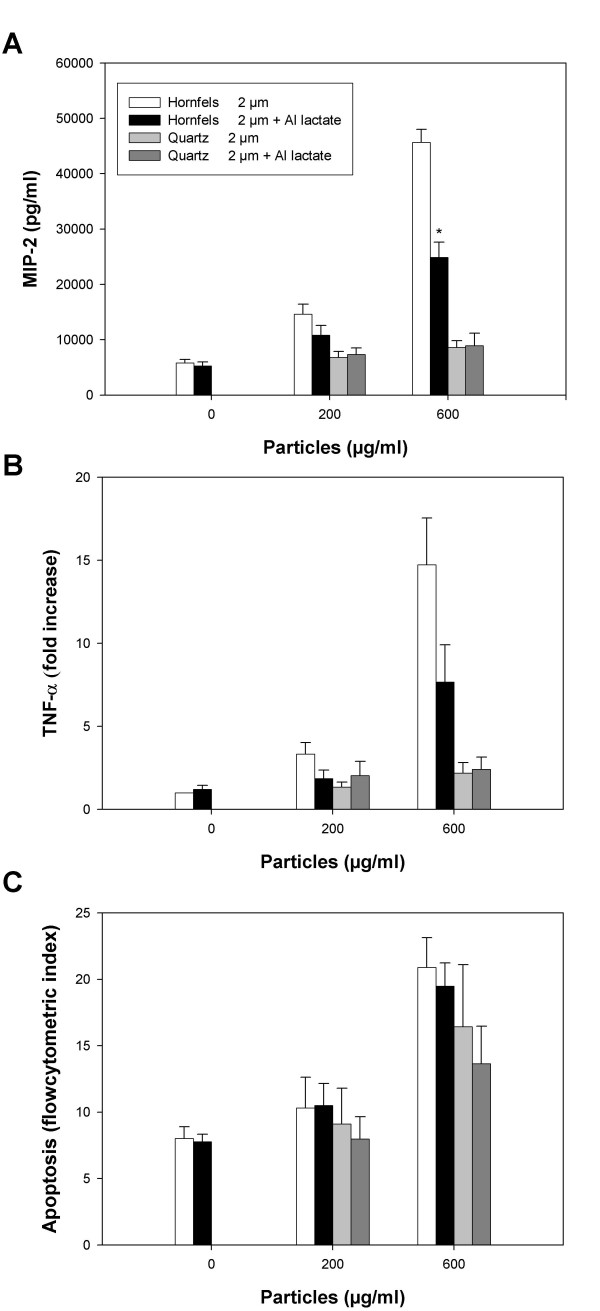
**The effect of surface modification of particles on MIP-2 release, TNF-α release and apoptosis**. **A) **MIP-2 release, **B) **TNF-α release and **C) **Apoptosis. Hornfels-2 and quartz-2 particles were treated in the absence or presence of aluminium lactate as described in Materials and Methods, and thereafter added to the cultured macrophages for 20 h. The MIP-2 and TNF-α release were analysed by ELISA, and apoptosis by flow cytometry after Hoechst 333258 staining. The results represent the mean +/- SEM of 3–5 independent experiments. *Significant reduction in MIP-2 release in aluminium lactate-coated versus non-coated hornfels-2 particles (p ≤ 0.05).

To further examine the importance of particle surface reactivity, particles were pre-treated with aluminium lactate. This compound is known to interact with quartz surface, resulting in a change in surface reactivity and reduced inflammatory activity [27]. Fig. 5 shows the effect of aluminium lactate pre-treatment on the cytokine release (TNF-α and MIP-2) and apoptotic responses induced by hornfels-2. The release of MIP-2 and TNF-α induced by the hornfels-2 particles was significantly reduced by the aluminium lactate treatment, whereas the apoptosis was not significantly affected. For comparison quartz-2 was treated similarly as hornfels-2. As for hornfels-2, aluminium lactate treatment did not alter apoptosis due to quartz-2. The minor cytokine response elicited by quartz-2 precluded the possibility to test the effect of aluminium lactate treatment on this response.

**Figure 6:**
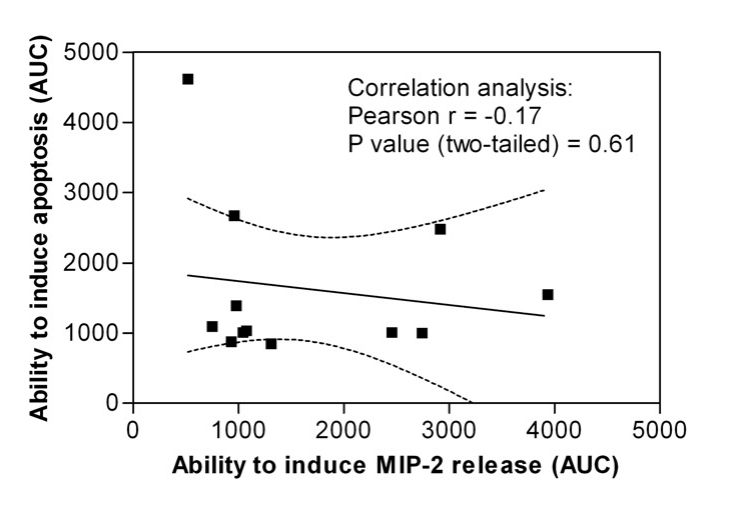
**Relationship between the particles abilities to induce MIP-2 release and apoptosis**. The figure displays the relative abilities of all the tested particles to induce MIP-2 release plotted against their abilities to induce apoptosis. The values are based on AUCs of relative increases in particle-induced MIP-2 release and apoptosis from the concentration-effect curves in Figs. 1A, 1E, 3A, 3C, 4B and 4D (concentration range: 0–600 μg/ml). Each point depicts the mean of 3–7 independent experiments. The figure also displays the result from a correlation analysis of the relative abilities to induce MIP-2 release versus apoptosis, as well as the linear regression line for the same data set with 95% confidence intervals.

To further examine the relationship between the particles abilities to induce cytokine release and their abilities to induce cell death, the correlation between MIP-2 release and apoptosis were analysed. Fig. 6 presents the relative abilities to induce MIP-2 plotted against their abilities to induce apoptosis of all the tested particles. As seen from the figure the two parameters were not correlated.

## Discussion

### Different particle characteristics are involved in triggering of cytokine responses and cell death

A crucial question in particle toxicology is whether the same particle characteristics govern different biological responses. Previous studies in our laboratory using lung epithelial cells *in vitro *and rats *in vivo *have shown that stone particles (≤ 10 μm) of various mineral compositions have different potentials to induce inflammatory responses [22,24,28]. To further elucidate this, rat alveolar macrophages were exposed to a wide range of varying concentrations of stone particles of different compositions and sizes, and examined for changes in cytokine release, apoptosis and cell viability. The results showed that the stone particles of different mineral compositions and sizes induced different response patterns for the end-points examined. Statistical analysis confirmed that there was no correlation between the ability of the particles to induce MIP-2 release and their ability to induce apoptosis. Thus it appears that different particle characteristics are involved in triggering of these cellular responses. In support of this, Elias and coworkers [29] have reported that different iron-containing minerals induce a different pattern for cytotoxicity and transforming effects in Syrian hamster embryo cells. For urban air particles (PM_2.5 _and PM_10_) varying particle characteristics were associated with cytotoxicity and cytokine responses [30]. Furthermore, deferoxamine, a chelator of transition metals, inhibited cytotoxicity, but not cytokine production induced by coarse urban particles, suggesting that the critical particle determinants and initial pathways for these cellular responses are different [31]. More recently, Huang and coworkers [32] addressed this question more systematically by comparing the relative ability of four different particles of quite diverse origin to induce apoptosis, necrosis and cytokine release in human alveolar macrophages. In accordance with our findings they found that the pro-apoptotic effects of particles were unrelated to their pro-inflammatory properties.

### Modulation of cytokine release was not due to cell death

Theoretically, the particle-induced changes in cytokine release in the macrophages could be due to changes in cell viability and apoptosis. However, our data do not indicate that the different cytokine responses induced by the particles of different sizes, can be attributed to differential reduction in cell viability. On a mass basis the MIP-2 response to quartz-0.5 was markedly stronger than for quartz-2, although the reduction in cell viability was approximately similar. Furthermore, when adjusted for particle surface areas only relatively small differences in cell viability were observed between small and large quartz particles. In contrast, the differences in the abilities to induce cytokine release were not abolished after adjusting for particle surface area (Fig. 1). These findings are supported by the experiments in Fig. 4, in which the stronger cytokine response to hornfels-2 than hornfels-10 is not reflected in a differential reduction in cell viability. It should be noted that at high concentrations of particles, at which the cytokine responses tended to decrease, a reduction in cell viability is presumably the explanation (Fig. 1).

With respect to apoptosis, quartz-2 seemed more potent than quartz-10 in the macrophages (Fig. 1, 4). Thus, it cannot be excluded that the slight cytokine response induced by quartz-2 exposure is partly due to apoptosis. In J774 macrophages, it has been hypothesized that the high capacity of Al_2_O_3 _particles to induce apoptosis might explain the low TNF-α release from these cells [33]. Conversely, it could be that the apoptosis observed in macrophages is induced by TNF-α. It has been shown that macrophages undergo apoptosis in response to TNF-α [34]. However, in the present study we could not observe any correspondence between the ability of the different particles to induce TNF-α release and induction of apoptosis (Fig. 4), suggesting that TNF-α is not a critical determinant in triggering the apoptotic process in the alveolar macrophages.

### Particle surface reactivity: more important for cytokine responses than cytotoxicity

An important observation in the present study is that for the examined stone particles/mineral particles surface reactivity seemed important for the ability to induce cytokine responses, whereas the surface reactivity seemed of less importance for the reduction in cell viability and apoptosis. Minor differences in apoptosis were observed for mylonite, gabbro, basalt, quartz, feldspar, hornfels and porphyr (≤ 10 μm). However, quartz-2 was more potent than hornfels-2 and porphyr-2 in inducing apoptosis. To further examine whether the surface composition was most crucial for the ability to induce cytokines, and contributed less to induction of apoptosis, the mineral particles hornfels-2 and quartz-2 were pre-treated with aluminium lactate. This treatment has previously been shown to reduce or abolish the particle surface reactivity of quartz [27,35]. In the present study the particle-induced apoptosis was not affected, whereas the particle-induced cytokine release was significantly diminished, indicating a role for the surface reactivity in the latter response. Previously, it has been established that mineral particle surface reactivity is critical for the ability to induce cytokine formation/inflammatory responses [22,24,26,35]. Somewhat in contrast to our findings, particle surface composition has also been reported to contribute to cytotoxicity/cell death, for example with quartz being more potent than titanium oxide [36,37]. Furthermore, surface modification of quartz (DQ12) particles by aluminium lactate inhibited the quartz-induced cytotoxicity, as measured by release of lactate dehydrogenase [38]. Notably, we have observed that for MinuSil, a standard quartz particle that is more potent to induce apoptosis/cell death than quartz-2 (Norquartz), aluminium lactate pre-treatment reduced the apoptotic response (unpublished results). Whether aluminium lactate only attenuates the apoptotic effect of particles with higher toxicity than our stone particles remains to be clarified.

In the present study it has not been examined which specific particle characteristics that are responsible for the cytokine responses and changes in apoptosis and cell viability. Previous findings from our laboratory suggest that insoluble components of the stone particles are driving the observed effects [22,25], but the critical particle components or characteristics still remain to be identified. Presumably, multiple factors or components are involved. Formation of reactive oxygen substances has been reported to mediate mineral particle-induced cytokine responses. However, in two previous studies, using many of the same particles as reported here, the results did not indicate that particle-derived ROS formation was driving neither cytokine release nor apoptosis [25,39]. Differential endotoxin content of the particles could be of importance, but this has not been analysed for these particles.

### The importance of particle surface area versus size for cellular responses

With respect to particle size/surface area and cellular responses, several *in vitro *studies have shown that mineral particles, polystyrene and carbon black induce inflammation/cytokines and cytotoxicity proportionally to their surface area [18-22,40]. Apoptosis and its relationship to particle size/surface area have received less attention. In our primary alveolar macrophages the picture was more complex, and could suggest that responses not only are attributed to surface area. Thus, for cytokine release (TNF-α, MIP-2), quartz-0.5 was more potent than the larger quartz particles, even after adjustment for particle surface area, and hornfels-2 was more potent than hornfels-10. For apoptosis quartz-2 seemed most potent, whereas the size-dependent differences between the effects of other particles were negligible. For quartz and other stone particles (hornfels, porphyr) of different sizes, the changes in cell viability were roughly similar after adjusting to particle surface area. The nature of the mechanisms involved in these effects is unclear, but it could be that particles of different sizes are recognized and taken up by the cells differently, and that this is of importance for the cellular outcome. In support of this, experiments with polyethylene and polystyrene particles in macrophages have shown that the small particles induced less cytokine responses than larger particles [41,42].

### Cell specific response patterns to particle exposure

Apparently, the response patterns depend on the cell types. Thus, in A549 cells exposed to quartz of the same size fractions as in the present study, the differences in cytokine release disappeared after particle surface area adjustment [19]. This is in contrast to the present data in the alveolar macrophages. The cell-specific pattern is also illustrated by the responses to microsilica. In the macrophages this particle type induced a marked apoptotic response and a reduction in cellular viability, but no cytokine release. Previously, we have observed that microsilica induced both a release of cytokines and a reduction of viability in A549 cells [19]. Thus, to elucidate the relative contribution of various particle determinants, it is important to compare the same end-points in the same culture system.

## Conclusion

Our data indicate that different mineral particle characteristics are critical for inducing apoptosis/cell death versus cytokine responses in primary rat alveolar macrophages. The surface reactivity of the mineral particles seems to be critical for cytokine responses in alveolar macrophages. In contrast, surface reactivity appeared less important for the ability of the types of tested particles to induce apoptosis. The cytokine responses and apoptosis did only partially correspond to particle surface area, suggesting that other mechanisms such as differential cellular recognition and uptake of particles of different sizes may contribute to the cellular responses. The data showing differential activation of end-points by different particle characteristics might be of importance for the risk assessment of particles. It might implicate that different end-points involved in various pathophysiological processes should be included when assessing the potential toxicity of ambient air particles using *in vitro *studies.

## Materials and methods

### Chemicals and reagents

RPMI culture medium and PenStrept were purchased from Bio Whittaker Europe, Verviers, Belgium. Foetal bovine serum (FBS) was from Gibco BRL, Paisley, Scotland. Ampicillin and fungizone were from Bristol-Myer Squibb, Bromma, Sweden. The enzyme-linked immunosorbent assay (ELISA) kits for MIP-2 and TNF-α (Cytoscreen, Cytoset) were from Biosource International, Camarillo, California, USA. Hoechst 33258 and 33342, Triton X-100, propidium iodide (PI), Nonidet P-40, Protein kinase K (KP0390), RNAase A (R5000) were obtained from Sigma Chemical Company, St.Louis, MO, USA. Aluminium lactate was from VWR International S.A.S, Fontanay sous Bois, France. All chemicals were of analytical grade.

### Particles and particle characterization

Different types of mineral particles, mylonite, gabbro, basalt, feldspar, hornfels, fine grain syenite porphyr (porphyr) and quartz, were provided and characterized for mineral composition, metal content, size distribution, and surface properties by SINTEF, NTNU, Trondheim, Norway (22, 24). For ≤ 10 μm fractions the median particle diameter was roughly similar, 6–8 μm. From the hornfels and porphyr ≤ 10 μm fractions, ≤ 2 μm fractions were produced. In addition, quartz particles (Norquartz-45 from Glamsland, Norway) were produced in size fractions ≤ 0.5 μm, ≤ 2 μm and ≤ 10 μm, with median diameters of 0.4 μm, 1.2 μm and 8.0 μm, respectively. The three size fractions of quartz were fractionated from the same batch. An amorphous silica, with a high iron content (Fesil microsilica from Hafslund Metall, Sarpsborg, Norway) used in the present study, had a median diameter of 0.3 μm [19]. The particles were prepared in FBS-free RPMI medium with antibiotics, at a concentration of 5 mg/ml, and sonicated in an ultrasonic water bath (Elma Ultrasonic T460) for 30 min and stored at 4°C. The particles samples used in the respective experiments were stored for the same time period (not more than 14 days), allowing a comparison between the samples. Particle suspensions were sonicated again for 30 min immediately before use.

With respect to aging of the particles the hornfels and porphyr particles were produced and the experiments conducted at a later time point than for the other particles. However, the data obtained within all the respective figures were performed with particles produced and fractionated in different sizes at the same time point, and should therefore be comparable. This does not exclude that freshly isolated particles would have been even more potent.

### Culture of rat alveolar macrophages

Male rats (WKY/NHsd) were purchased from Harlan, UK. The animals weighed 200–250 g at the time of sacrifice, and were given Ewos standard pelleted laboratory chow from Astra Ewos AB, Södertälje, Sweden and water *ad libitum*. Alveolar macrophages were obtained by lung lavage [43]. The macrophages were suspended in RPMI medium with ampicillin (0.1 mg/ml), penicillin (0.1 mg/ml), streptomycin (0.1 mg/ml), fungizone (0.25 μg/ml) and 5% heat-inactivated fetal bovine serum (FBS) at a cell density of 1.5 × 10^6 ^cells/ml and added to 35-mm Costar wells (1 ml/well). Non-attached cells were removed after 1 h, and fresh RPMI medium with FBS was added to the attached cells.

### Exposure to particles and study design

The alveolar macrophages were exposed to the particles of different sizes and chemical composition in 1.0 ml medium. After 8 or 20 h 50 μl of the cell culture medium was removed, and analysed for MIP-2 and/or TNF-α by enzyme-linked immunoabsorbent assay (ELISA). In parallel, the cells were examined for changes in viability and apoptosis as measured by flow cytometry, fluorescence microscopy and by DNA-laddering, respectively. The apoptotic potential was verified by DNA-laddering/fragmentation for some particles of different sizes. In separate experiments, the quartz-2 and hornfels-2 particles were suspended at a concentration of 5 mg/ml in a 1% solution of aluminium lactate in destilled water, to diminish the particle surface activity as described by Duffin and coworkers [27]. The particles were sonicated for 5 min, and agitated for at least 3 h at room temperature. Particles suspended in the presence of destilled water and treated in the same manner were used as controls to determine the effect of the coating procedure. After treatment with aluminium lactate or water, the particles were washed twice with saline (0.9% NaCl) by centrifugation at 2500 × g for 10 min to remove unbound aluminium lactate. Coated and non-coated particles were added to the cells, and changes in apoptosis and cytokine release were assessed after 20 h.

### MIP-2 and TNF-α assays

After exposure for 8 or 20 h supernatants were sampled and stored at -70°C. The release of MIP-2 after 20 h and TNF-α after 8 h (Fig. 4) and 20 h (Fig. 5) was quantified using ELISA according to the recommendations in the manufacturer's manual. Increase in colour intensity was quantified using a plate reader (TECAN Sunrise) with software (Magellan V1.10). The relative abilities of all the tested particles to induce MIP-2 release were quantified by estimating the area under the curve (AUC) of the concentration-effect curves (0–600 μg/ml) by use of GraphPad Prism software.

### Apoptosis measurement by flow cytometry

After exposure to particles for 20 h detached cells in the macrophage cultures were removed, the attached cells were trypsinized and combined with the respective detached cells. The apoptosis was determined using flow cytometry. The DNA of the macrophages was stained by incubating 0.5–1.0 × 10^6 ^cells in phosphate-buffered saline (PBS) containing 0.1% Triton X-100 and Hoechst 33258 (1.0 μg/ml) for 15 min in the dark. The DNA histograms were recorded on a Skatron Argus 100 flow cytometer (Skatron, Tranby, Norway) and analyzed using the Multiplus Program (Phoenix Flow systems, San Diego, CA, USA). Cell cycle phases and apoptotic cells/bodies were distinguished by their DNA content (Hoechst fluorescence) and cell size (forward light scatter). Apoptosis was defined as the registered counts/signals left to the G1 peak, with background subtracted. This background was set to approximately 20% of the G1 channel number. We usually observe the G1 peak at channel 100, and the background at channel 20 or below. The apoptotic index was determined as percentage of counts/signals in the area between the G_1 _peak and the background, relative to the total area excluding background and aggregates. The relative abilities of all the tested particles to induce apoptosis were quantified by estimating the AUC of the concentration-effect curves (0–600 μg/ml) by use of GraphPad Prism software.

### Apoptosis measured by DNA fragmentation assay

A549 cells were exposed to particles (quartz-2, quartz-10 and microsilica) at different concentrations in 35 mm Costar wells for 20 h. DNA fragmentation was performed according to the method of Gorczyca et al. [44]. Briefly, harvested cells (1.0 × 10^6^) were washed in PBS, resuspended in 0.25 ml of TBE (45 mM Tris borate buffer, 1 mM EDTA, pH 8.0) containing 0.25% Nonidet P-40 and 0.1 mg/ml RNAase and incubated at 37°C for 30 min. Proteinase K (1.0 mg/ml final concentration) was added and the samples were incubated for additional 30 min, prior to addition of 50 μl loading buffer (0.01 ml 0.1 M Tris, pH 7.5; 0.04 ml 0.5 M EDTA, pH 7.5; 0.5 ml glycerol (85%); 0.8 mg bromophenol blue and H_2_O to 1.0 ml). The samples were incubated at 65°C for 10 min immediately prior to application to the agarose gel (1.5%). The DNA bands were visualised under UV light in gels run with Gelstar, as described by the manufacturer.

### Fluorescence microscopic determination of cell viability and apoptosis

A549 cells were exposed to particles of different composition and sizes for 20 h. Cell viability and apoptosis was determined after staining cells with PI (5.0 μg/ml) and Hoechst 33342 (10 μg/ml) for 30 min in the dark. Briefly, the cells were centrifuged at 250 × g at 4°C for 10 min and washed twice. Smears on slides made from the pelleted cells suspended in FBS, were quickly air-dried. Cell morphology was evaluated using a Nikon Eclipse E 4000 microscope (original magnification × 1000). The cell viability was determined by the ability of the cells to exclude PI. Approximately 400 cells were counted on the smears. Apoptotic cells were identified by their distinct condensed nuclei and/or nuclear fragmentation. The identification of apoptotic cells were only done at low particle concentrations. When analysed by fluorescence microscopy it was difficult to identify the apoptotic cells due to coverage of the cells by the mineral particles. At low concentrations of mineral particles we observed similar levels of apoptosis by the two methods used (data not shown).

### Statistical analysis

Statistical comparisons were carried out using one-way ANOVA with Bonferoni multiple comparison test (p < 0.05). Correlation and linear regression analysis were performed with GraphPad Prism Software.

## Competing interests

The author(s) declare that they have no competing interests.

## Authors' contributions

MR designed and coordinated the experimental work of this study, interpreted the results and was mainly responsible for the writing of the manuscript. RBH performed several of the experiments, and was involved in the writing process. JØ performed a part of the experiments, and participated in critical assessment and in the writing process. IS participated partially in the study design and did the initial experimental work as a part of her master thesis. PES participated in critical assessment and writing of the manuscript. ML participated in study design, supervision of the experimental work, interpretation of the results, and writing of the manuscript. All authors have read and approved the manuscript.
